# Role of bone-anabolic agents in the treatment of breast cancer bone metastases

**DOI:** 10.1186/s13058-014-0484-9

**Published:** 2014-12-31

**Authors:** Attaya Suvannasankha, John M Chirgwin

**Affiliations:** 1 Department of Medicine, Indiana University School of Medicine, 980 W. Walnut Street, Walther Hall, Indianapolis R3-C312 USA; 2 Richard L. Roudebush, VA Medical Center, Indianapolis, 46202 IN USA

## Abstract

Skeletal metastases are an incurable complication afflicting the majority of patients who die from advanced breast cancer. They are most often osteolytic, characterized by net bone destruction and suppressed new bone formation. Life expectancy from first diagnosis of breast cancer bone metastases is several years, during which time skeletal-related events - including pain, fracture, hypercalcemia, and spinal cord compression - significantly degrade quality of life. The bone marrow niche can also confer hormonal and chemo-resistance. Most treatments for skeletal metastases target bone-destroying osteoclasts and are palliative. Recent results from the Breast cancer trials of Oral Everolimus-2 trial suggest that agents such as the mammalian target of rapamycin inhibitor everolimus may have efficacy against breast cancer bone metastases in part via stimulating osteoblasts as well as by inhibiting tumor growth. Selective estrogen receptor modulators similarly inhibit growth of estrogen receptor-positive breast cancers while having positive effects on the skeleton. This review discusses the future role of bone-anabolic agents for the specific treatment of osteolytic breast cancer metastases. Agents with both anti-tumor and bone-anabolic actions have been tested in the setting of multiple myeloma, a hematological malignancy that causes severe osteolytic bone loss and suppression of osteoblastic new bone formation. Stimulation of osteoblast activity inhibits multiple myeloma growth - a strategy that might decrease breast cancer burden in osteolytic bone metastases. Proteasome inhibitors (bortezomib and carfilzomib) inhibit the growth of myeloma directly and are anabolic for bone. Drugs with limited anti-tumor activity but which are anabolic for bone include intermittent parathyroid hormone and antibodies that neutralize the WNT inhibitors DKK1 and sclerostin, as well as the activin A blocker sotatercept and the osteoporosis drug strontium ranelate. Transforming growth factor-beta inhibitors have little tumor anti-proliferative activity but block breast cancer production of osteolytic factors and are also anabolic for bone. Some of these treatments are already in clinical trials. This review provides an overview of agents with bone-anabolic properties, which may have utility in the treatment of breast cancer metastatic to the skeleton.

**Electronic supplementary material:**

The online version of this article (doi:10.1186/s13058-014-0484-9) contains supplementary material, which is available to authorized users.

## Introduction

Almost 40,000 women die from advanced breast cancer yearly in the US, the majority with bone metastases; 85% of them will have bone-destructive (osteolytic) skeletal lesions, which cause hypercalcemia, fracture, severe and intractable bone pain, and nerve compression. Average survival from time of diagnosis of bone metastasis is 2 to 3 years, and about 10% of women with breast cancer already have metastases when first diagnosed [1]. Osteolytic metastases are characterized by not only bone destruction but also the inhibition of normal formation of new bone, worsening the skeletal insult caused by metastatic tumor [2]. While breast cancer therapy focuses largely on tumor cells, agents that target bone may not only reduce skeletal-related events but also sensitize the tumor to conventional therapies. The hematological malignancy, multiple myeloma (MM), though very different from breast cancer, also colonizes and attacks the skeleton. Both tumor types, when lodged in the skeleton, stimulate osteolytic bone destruction. Several classes of agents against myeloma have actions on the osteoblast lineage and might be useful against osteolytic metastases in advanced breast cancer. Data are lacking that bone-biosynthetic osteoblasts oppose breast cancer growth in bone, but such a mechanism is documented in MM. The potential application to breast cancer of agents with bone-anabolic activity is the focus of this review.

### Osteolytic bone metastases can be modeled as a vicious cycle

**Figure 1:**
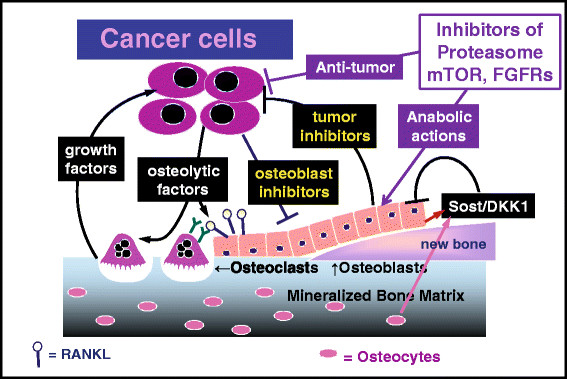
**Vicious cycles in bone metastasis.** In the classic vicious cycle of bone metastasis [2], tumor cells stimulate osteolysis by releasing factors (such as interleukins and parathyroid hormone-related protein) that increase receptor activator of nuclear factor kappa-B (RANK) ligand (RANKL, shown as lollipops), which activates osteoclasts (by binding to the RANK receptor, shown as Y’s) to resorb bone. Bone resorption releases growth factors from matrix (such as transforming growth factor beta), which in turn stimulate tumor cells. In multiple myeloma (MM), an additional vicious cycle occurs. Active osteoblasts make new bone and secrete factors that antagonize tumor growth. MM and breast cancer cells secrete osteoblast blockers, such as sclerostin (Sost) and DKK1 (both inhibitors of the WNT pathway), thus potentially fending off osteoblast-mediated growth inhibition and causing increased bone loss, because tumor now increases osteoclastic bone destruction and suppresses compensatory new bone formation. The authors speculate that a similar secondary vicious cycle occurs in osteolytic bone metastases because of breast cancer. The role of abundant osteocytes in bone metastasis is unclear, although these cells are a major source of sclerostin and RANKL. FGFR, fibroblast growth factor receptor; mTOR, mammalian target of rapamycin.

Osteolytic bone metastases can be modeled as a vicious cycle (Figure 1), in which tumor cells stimulate bone destruction via osteoclast activation, releasing active growth factors from bone matrix, which in turn stimulate tumor growth [2]. Bone is resorbed by rare cells of the hematopoietic lineage, multinucleated osteoclasts, whose formation is controlled by the factor receptor activator of nuclear factor kappa B ligand (RANKL), made by cells in the osteoblastic lineage, including abundant osteocytes embedded within mineralized bone matrix [3]. Tumor cells stimulate bone production of RANKL, which can be neutralized by osteoprotegerin (OPG) also made by bone cells [4]. A pathologically increased RANKL/OPG ratio results in net bone loss. Osteoclasts are the major targets of current bone-specific palliative therapies for skeletal metastases, including bisphosphonates and the RANKL-neutralizing monoclonal antibody, denosumab [5]. Osteoclast-targeted therapies are a mature frequently reviewed field and not discussed here, since the available agents are highly effective and unlikely to be further improved. Targeting osteoclasts alone, though it blocks bone destruction, is insufficient to restore skeletal integrity, leaving patients at risk for fracture even during disease remission. Bone loss is further increased by anti-estrogen therapy for hormone receptor-positive breast cancer. Hence, we focus on drugs (approved or in clinical development) with stimulatory actions on cells of the osteoblast lineage.

### Roles of bone cells in normal bone remodeling

Bone is a dynamic tissue that is slowly remodeled by the action of osteoclasts to remove packets of old bone, followed by the synthesis of new bone by osteoblasts. The osteoblast lineage is complex and incompletely understood. Mesenchymal precursors differentiate into early, proliferative osteoblasts, which mature into late biosynthetic osteoblasts that lay down new bone. Some cells of this lineage continue to differentiate into osteocytes: these are long-lived cells embedded within bone matrix and connected to one another and the bone surface via intracanalicular processes. Osteocytes are the most abundant bone cell type and as yet little studied in cancer/bone diseases. They are the major source of several endocrine factors secreted by bone, including sclerostin and fibroblast growth factor (FGF)23 [6]. In healthy individuals, bone formation and resorption are kept in long-term balance by poorly understood coupling mechanisms. Osteoporosis is the consequence of a modest net excess of resorption over formation. Bone resorption is elevated in all types of cancer/bone disease [7]. Osteolytic lesions occur in 85% of breast cancer bone metastases and in MM. Osteoblastic/osteosclerotic bone metastases occur in prostate cancer and in about 15% of breast cancer patients with skeletal metastases [2]. Loss of the balance between formation and resorption suggests that coupling is disrupted by the presence of tumor through unknown mechanisms. Complicating the issue is that cells of the osteoblast lineage are the source of RANKL, OPG, and the WNT inhibitor sclerostin. It is unlikely, however, that biosynthetic late osteoblasts, which lay down new bone, are the source of these factors.

### Markers of bone resorption and formation

Biochemical markers of bone metabolism are either bone cell-secreted products (such as alkaline phosphatase from early osteoblasts and osteocalcin from late osteoblasts) or metabolites of collagen formation and degradation. Both resorption markers and alkaline phosphatase are increased in most patients with cancer in response to treatments, many of which cause drug-induced osteoporosis [7]. Thus, bone markers do not provide information about changes in osteoblast function in bone metastases, which are inferred from laboratory experiments rather than clinical data.

### Evidence for suppression of osteoblasts by tumor cells

Osteoblasts are very difficult to identify histologically, even when bone biopsies are available. Thus, the data for osteoblast suppression, even in MM, are derived mostly from observations *in vitro* rather than from the clinic. Tumor suppression of osteoblasts is seen *in vitro* and may be due to production by breast cancer cells of IL-1 and IL-11, tumor necrosis factor-alpha, platelet-derived growth factor, or Fas ligand or indirectly due to osteoclastic activation of transforming growth factor-beta (TGFβ) [8],[9].

### Restoration of lost bone is a major goal of osteoporosis treatment

Restoration of lost bone is a major goal of osteoporosis treatment, to which end a variety of bone-anabolic agents, including once-a-day parathyroid hormone (PTH) and strontium ranelate, have been developed. For the purposes of this discussion, we define anabolic agents as those that stimulate biosynthetic osteoblasts to synthesize new bone, rather than drugs that stimulate all parts of the long and complex osteoblast lineage. Several anti-tumor agents have positive effects on bone health: proteasome inhibitors (PIs) (bortezomib and carflizomib) are US Food and Drug Administration (FDA)-approved for MM therapy [10]. Mammalian target of rapamycin (mTOR) inhibitors have osteoblast-stimulatory as well as osteoclast- and tumor-inhibitory actions [11]. Proof-of-principal for the value of osteoblast stimulation in the treatment of osteolytic breast cancer metastases was suggested by exploratory studies of bone parameters (reviewed [11] in the BOLERO-2 (Breast cancer trials of Oral Everolimus-2) trial (exemestane + the mTOR inhibitor everolimus) [12].

### Selective estrogen receptor modulators

Selective estrogen receptor modulators (SERMs) (such as tamoxifen and raloxifene) have anti-tumor activity against estrogen receptor-positive (ER^+^) breast cancers while also stimulating bone formation. Promising agents with bone-anabolic actions that do not directly inhibit tumor proliferation include TGFβ inhibitors and neutralizing antibodies against the WNT inhibitors DKK1 and sclerostin (which are negative regulators of bone formation). This review discusses the applicability of these agents for breast cancer bone metastasis.

## Review

The role of anti-resorptive agents in the treatment of bone metastases is well established. In addition to tumor-induced osteolysis, conventional cancer treatments stimulate osteolysis, often by suppression of sex steroid activity [13],[14]. Some anti-cancer agents also have direct effects to increase osteoclast activity, such as geldanamycin derivatives, which make osteolytic metastases worse unless combined with an anti-resorptive agent [15]. Bone marrow provides a major tumor stem cell niche, which may be altered in states of high bone turnover [16]. In a number of animal models, experimental stimulation of bone turnover increases skeletal metastases, suggesting that high bone turnover is generally undesirable in cancer and should be opposed with anti-resorptive drugs.

Inhibitors of osteoclastic bone resorption are the standard of care for all cancers growing in bone, whether the overall bone response is osteolytic or osteoblastic [5]. Available agents fall into three classes: cathepsin K inhibitors, bisphosphonates, and RANKL-neutralizing antibody. Cathepsin K is a lysosomal enzyme with activity that is essential during bone resorption by active osteoclasts [17]. Bisphosphonates, such as zoledronic acid, are metabolic poisons that bind avidly to mineral surfaces in bone, whence they are ingested by active osteoclasts, which they then kill. RANKL is expressed by cells in the osteoblast lineage and controls the differentiation of osteoclasts from hematopoietic precursors as well as their activity and survival [4]. Neutralization of RANKL with the monoclonal antibody denosumab effectively blocks the formation of osteoclasts. Denosumab appears to be the most effective of the anti-resorptive agents. Some anti-cancer drugs can inhibit osteoclasts, but they have limited additional benefit in patients already receiving potent anti-resorptive agents.

### mTOR inhibitors and lessons from the BOLERO-2 trial

The phosphoinositide-3-kinase-Akt-mTOR pathway is a key mediator of cellular proliferation, apoptosis, migration, and angiogenesis - all critical to tumor aggressiveness [18]. It is commonly activated in breast cancer, conferring resistance to hormonal therapy and trastuzumab. Blockade of the pathway at multiple levels overcomes resistance, and inhibitors are in clinical development. The mTOR inhibitors rapamycin, everolimus, and temsirolimus are of particular interest for breast cancer bone metastasis because they have positive actions on bone in addition to sensitizing tumors to hormonal therapy and trastuzumab. mTOR inhibition suppresses RANKL and cathepsin K and increases OPG secretion by bone marrow stromal cells [19]. Everolimus promotes osteoblast differentiation and decreases bone loss associated with estrogen deprivation in breast cancer models [20]. BOLERO-2 is a phase III trial of everolimus plus the steroidal aromatase inhibitor (AI) exemestane versus placebo plus exemestane in women with metastatic ER^+^ breast cancer recurring or progressing despite AI therapy [12]. The everolimus arm showed a superior response rate (7% versus 0.4%) and progression-free survival (10.6 versus 4.1 months), as well as lowered bone resorption markers and decreased bone progression across subgroups, regardless of bisphosphonate use and baseline bone metastases. Both everolimus-mediated sensitization to exemestane and its bone effect may contribute to the positive results of the combination. The relative contribution of each mechanism cannot be quantified in clinical studies, but an early reduction in bone turnover markers prior to clinical response and the reduced bone complications in the everolimus arm support bone targeting as a therapeutic strategy in breast cancer [11]. Whether everolimus-mediated bone benefit can decrease future bone metastases will be answered by an ongoing clinical trial exploring everolimus in an adjuvant setting (ClinicalTrials.gov identifier: NCT01674140). The benefits from everolimus point the way to new clinical trials to test other novel agents with positive effects on bone, including the src inhibitor desatinib, the c-met and vascular endothelial growth factor receptor (VEGFR)2 inhibitor cabozantinib, and the phosphoinositide-3-kinase inhibitor BKM120 in bone metastases due to breast, prostate, and renal cancers.

### Proteasome inhibitors

The proteasome degrades damaged, misfolded, and short-lived proteins. Cancer cells are vulnerable to metabolic stress caused by blockade of this pathway. Although high proteasome activity is seen in many cancer types, PIs are particularly cytotoxic to MM and mantle cell lymphoma. Two PIs, the first-in-class boronate peptide bortezomib and the epoxyketone carfilzomib, are FDA approved for myeloma treatment. Other anti-myeloma agents improve bone health by killing MM cells, thereby reducing cancer-induced osteoclastogenesis. PIs have additional, bone-anabolic actions, suggesting their potential for use against osteolytic solid tumor bone metastases. Bortezomib and carfilzomib inhibit osteoclast formation and bone resorption while enhancing osteoblastic differentiation and mineralization *in vitro* [21],[22]. Carfilzomib and its orally active analog oprozomib increase trabecular bone volume and decrease bone resorption in normal and MM-bearing mice [23]. In patients with MM, bortezomib and carfilzomib reduce markers of bone resorption but also increase those of bone formation (alkaline phosphatase and osteocalcin) regardless of tumor response, suggesting direct bone-anabolic effects [24],[25].

The detailed mechanisms of the bone-anabolic actions of PIs remain unclear but may result from enhanced WNT/β-catenin signaling in osteoblasts due to decreased DKK1 [26]. Bortezomib is cytotoxic for breast cancer cells *in vitro* and suppresses tumor growth and osteolysis when breast cancer cells are directly injected into the tibiae of mice [27]. In clinical trials, bortezomib had marginal single-agent efficacy against advanced disease, but bone metastases were not analyzed in these studies [28]. Bortezomib has additive cytotoxicity when used in combination with a broad array of current agents (such as doxorubicin and paclitaxel) or those in development for breast cancer (such as SAHA, Hsp90 inhibitors, and lapatinib). Common chemotherapeutics generally increase bone loss [7]. Incorporation of PI in a breast cancer treatment regimen could enhance tumor killing, prevent further tumor growth in bone, and improve bone health - an approach worthy of clinical development.

### Anti-tumor effects of osteoblasts

Data from patients with MM, experiments in animals, and co-cultures of MM cells with bone cells all suggest that osteoblasts and their products oppose growth of MM cells in bone. This suggests a second vicious cycle in cancer-bone interactions (Figure 1), in which MM and other osteolytic cancers suppress osteoblasts to overcome the growth-inhibitory effects of osteoblasts on tumor. The marker of osteoblast activity, alkaline phosphatase, is suppressed in MM, and co-culture with osteoblasts inhibits MM cell growth, whereas their co-culture with osteoclasts has the opposite effect [29]. The data suggest a model in which osteoblast-secreted products, such as decorin, are locally and specifically cytotoxic for MM cells. Similar data exist in breast cancer. When breast cancer cells were cultured on a bone substrate containing osteoclasts, the introduction of osteoblasts decreased bone resorption [30]. This effect is the opposite of what would be expected if osteoblasts functioned primarily as the source of RANKL. Osteoclasts, on the other hand, catalyze the release of a panoply of growth factors from their immobilized storage site within bone matrix (Figure 1), which stimulate breast cancer and MM growth, driving the basic vicious cycle. Complicating the issue is the regulation of osteoclast formation by RANKL, which is expressed by subsets of cells in the multi-stage osteoblastic lineage. Thus, a drug that stimulated RANKL^+^ cells in the osteoblast lineage could increase rather than decrease osteolysis and tumor growth.

One bone-anabolic agent is currently widely used in the treatment of osteoporosis: once-daily injection of PTH 1-34 (teriparatide). When given to experimental animals, teriparatide increased new bone formation and resulted in suppression of myeloma growth [31]. The authors proposed that osteoblasts were stimulated to secrete anti-myeloma factors. Identification of such factors would facilitate development of more selective anti-myeloma treatments that might also be effective against bone metastases due to solid tumors, including breast cancer. The authors isolated myelomatous bone from mice treated with PTH or saline for 4 weeks and examined RNA for changes in gene expression by array hybridization. PTH increased many markers of osteoblast activity, such as collagens and osteocalcin, but also altered members of the WNT signaling pathway, including reducing DKK1 mRNA, but not sclerostin. Teriparatide carries a black box warning against its use in patients with cancer, due to an increase in osteosarcomas in rats treated with high doses of PTH, and is unlikely to be approved for use in oncology. Nonetheless, the anti-tumor effects of PTH provide proof-of-principal for the use of bone-anabolic agents against myeloma bone disease and osteolytic breast metastases. The anabolic actions of drugs can be tested on bone separately from their effects on tumor growth, as has been done for proteasome inhibitors and MM. However, it is very difficult to evaluate the relative contributions to anti-tumor efficacy of a multi-tasking agent with both direct, anti-tumor and indirect, bone-anabolic actions. A potent bone-anabolic agent without direct actions on breast cancer growth might be useful against bone metastases in the clinic.

### Roles of osteocytes

The osteoblastic lineage is complex, spanning many weeks and phenotypes. Cells in the lineage not only lay down new bone, they regulate osteoclasts via RANKL/OPG, while late osteoblasts can become osteocytes, the cells immured in mineralized matrix and interconnected by dendritic processes running in a canalicular network within bone. Osteocytes are very long-lived and the most abundant cell type in bone, compared with osteoblasts and rare osteoclasts. Because of their entrapment in bone matrix, they are difficult to study in conventional tissue culture; however, they are now known to make RANKL and to be the major site of sclerostin production. Osteocytes are resistant to apoptosis, but their numbers are decreased in bone chronically exposed to myeloma cells. Maturation of late osteoblasts into osteocytes may be blocked by factors made by MM or breast cancer, or osteocytes may be reprogrammed by the local presence of tumor cells. MM cells can cause osteocyte apoptosis and also contribute to increased osteolysis by stimulating osteocyte production of IL-11 [32].

### Selective estrogen receptor modulators and negative effects on bone of aromatase inhibitors

SERMs interact with ERs in target organs as either ligand agonists or antagonists. The main anti-estrogen treatments are SERMs, such as tamoxifen, and third-generation AIs, such as exemestane, letrozole, and anastrozole. AIs have replaced tamoxifen in the past decade in the adjuvant setting because of the lack of thromboembolic and uterine cancer side effects and improved disease-free survival. Over 80% of breast cancers are ER^+^. Therefore, these agents have been extensively used at various stages of breast cancer from prevention in high-risk populations to neoadjuvant therapy in advanced-stage disease, both alone and in combination with chemotherapy. However, AIs reduce bone mineral density (BMD) and increase fractures, whereas tamoxifen preserves BMD in postmenopausal women [33]. The effect of AIs is only partly attenuated by bisphosphonates. Mortality rate at 3 months after hip fracture is higher with age [34], suggesting that some patients treated with AIs will die from complications of therapy and not from cancer. The data highlight the possible long-term harm of cancer therapy and the need to identify patients at highest risk for bone complications as well as to develop strategies to overcome excessive bone loss associated with AIs.

### Other agents with possible anabolic actions

Strontium ranelate effectively reduced osteoporotic vertebral fractures in clinical trials, whereas *in vitro* it can inhibit bone resorption and stimulate bone formation. The clinical data are contradictory and the cellular mechanisms unclear [35]; the agent was recently found to increase the relative risk of myocardial infarction and is unlikely to be approved for clinical use in the US. Substitution of Sr for Ca in bone mineral is the basis for using the beta emitter ^89^Sr for the palliation of metastatic bone pain - an application different than that of the non-radioactive element. Sr may be an anti-fracture agent and not a bone-anabolic agent but might still be effective against bone metastases [36].

### Tyrosine kinase inhibitors

Cabozantinib inhibits multiple receptor tyrosine kinases, including MET and VEGFR2; it has direct anti-tumor activity and may also stimulate normal bone formation in patients with advanced prostate cancer [37]. A number of pan-fibroblast growth factor receptor (FGFR) kinase inhibitors are in clinical trials [38], and one, NVP-BGJ398, enhanced bone growth and mineralization in a mouse model [39]. Pan-FGFR inhibitors were originally expected to be effective against tumors with specific FGFR overexpression (and hence *addicted* to FGFR signaling). More recently, pan-FGFR inhibitors have been brought into clinical trials for tumors without FGF-signaling addiction. Such inhibitors - and multi-kinase inhibitors that share anti-pan-FGFR activity, such as dovitinib, nintedanib, and ponatinib - may also have value for the specific treatment of breast cancer bone metastases by adding bone-anabolic actions to direct tumor inhibition.

### Inhibitors of transforming growth factor beta superfamily members

The TGFβ superfamily of over 30 ligands includes TGFβs, activins, and bone morphogenetic proteins (BMPs). The ligands signal through a complex array of heterodimeric serine/threonine kinase receptors that activate intracellular Smad signaling pathways [40]. Members of each family within the superfamily are active in cancer and bone, and several have well-characterized effects on bone anabolism.

### Activin A

Activin A is a multifunctional cytokine of the TGFβ superfamily. It contributes to a broad range of cellular pathways, including bone homeostasis, bone marrow erythropoiesis, and skeletal muscle mass. It is abnormally regulated in cancers, including breast, prostate, ovarian, and hepatocellular carcinoma, and has been associated with tumor aggressiveness [41]. Activin A signals through the type 2A receptors on bone cells (both osteoblasts and osteoclasts) and stromal cells. Its role in both bone resorption and bone formation suggests that inhibitors would be bone-anabolic agents. Vallet and colleagues [42] found that activin A was elevated in both the bone marrow and peripheral blood of patients with MM. Co-culture with MM cells increases activin A in bone marrow stromal cells and osteoclasts. Terpos and colleagues [43] correlated activin A to extent of bone involvement and poor survival in patients with MM. Activin A receptor antagonists increase bone formation in monkeys and in postmenopausal women as well as prevent development of MM bone lesions and decrease tumor burden in a murine model of MM, suggesting that they enhance bone formation *in vivo* [42],[44]. A recently completed phase II randomized trial of sotatercept (ACE-011), a soluble activin receptor type 2A IgG-Fc fusion protein, in MM patients with osteolytic lesions (ClinicalTrials.gov identifier NCT00747123) showed increased bone-specific alkaline phosphatase and decreased bone resorption marker C-terminal telopeptide (CTX), when used with melphalan and prednisone, compared with melphalan + prednisone controls, in bisphosphonate-naïve patients. Sotatercept also promotes red blood cell production and therefore was evaluated in a phase II trial of patients with chemotherapy-induced anemia and metastatic breast cancer [45]. Although improvement of hemoglobin and a favorable toxicity profile were observed, bone was not included as an endpoint in the study. A similar fusion protein increased bone formation and inhibited growth of breast cancer and MM in bone in preclinical models [46]. The benefit of activin A antagonists against solid tumor bone metastases certainly warrants further evaluation.

### Transforming growth factor beta inhibitors

TGFβ is a two-edged sword in cancer. It is a tumor suppressor early in tumorigenesis, but breast cancers progress to escape its growth inhibitory effects and respond to TGFβ by increasing expression of prometastatic genes [47]. Mineralized bone matrix is the major store of immobilized TGFβ in the body, whence it is released and activated by osteoclastic bone resorption; TGFβ thus plays a specific and important role in bone metastases as well as in normal bone metabolism. Inhibition of TGFβ with neutralizing antibodies or receptor kinase inhibitors is effective against animal models of breast cancer bone metastases and may reach the clinic [47]. Selective small-molecule inhibition of the TGFβ type I receptor kinase is anabolic for bone while suppressing osteoclasts [48], suggesting that such inhibitors may have triple actions to inhibit tumor and osteoclasts while stimulating osteoblasts.

A downstream mediator of the effects of TGFβ is tumor-secreted Jagged1, which activates Notch signaling on bone stromal cells to increase pro-osteolytic IL-6 - a process that can be blocked with a γ-secretase inhibitor [49], but it is not known whether γ-secretase inhibitors have bone-anabolic activity.

### WNT inhibitor blockers

BMP and WNT pathways are major regulators of normal osteogenesis. WNT signaling is inhibited by specific antagonists - sclerostin, DKK1, and secreted frizzled-related proteins [50] - resulting in reduction of new bone formation to basal levels. All three inhibitory proteins can be made by tumors and bone and may contribute to osteoblast suppression [51]. Breast cancer cells make sclerostin and DKK1, whereas serum DKK1 is increased in patients and contributes to osteolytic bone destruction in breast cancer bone metastases [52]. Production of sclerostin by breast cancer cells blocks osteoblast differentiation [53]. Although the WNT pathway is active in most tumor cells, the WNT antagonists show specific effects on bone, where they depress bone formation to a basal level rather than blocking it entirely. DKK1 is made by MM cells and increased in patient sera. A Dkk1-neutralizing antibody is in clinical trials for MM [54], and sclerostin-neutralizing antibodies have been developed for osteoporosis [55]. Neutralization of WNT inhibitors might be effective against breast cancer bone metastases.

### Summary of potential agents for bone metastases with bone-anabolic activity

#### US Food and Drug Administration approved agents

The mTOR inhibitor everolimus is in use in the US for metastatic breast cancer, following the BOLERO-2 trial. Other mTOR inhibitors are FDA approved for other indications and may have similar effects. SERMs such as tamoxifen and raloxifene are approved but have been largely superseded by AIs with their superior effects on overall survival. PIs are approved in myeloma and have been in clinical trials in breast cancer, although bone metastases were not included [28]. On the basis of the strong preclinical results from the Lian laboratory [27], bortezomib merits further clinical trials in breast cancer with inclusion of bone metastases.

#### Agents in clinical trials

Agents in clinical trials include drugs that neutralize activin A or TGFβ or block their receptors. Many tyrosine kinase inhibitors may have bone-anabolic activities. We will know whether these agents are useful against bone metastases only if breast cancer trials enroll patients with confirmed bone metastases. Where such data are lacking, candidate agents need to be tested in preclinical models of breast cancer bone metastases to inform the design of clinical trials to include endpoints for bone morbidity and tumor growth in bone. Antibodies that neutralize DKK1 or sclerostin are in trials [54],[55] and may have beneficial effects to relieve the suppression of bone formation caused by these WNT inhibitors, which can be made by breast cancer cells and myeloma as well as by bone (osteocytes in the case of sclerostin).

#### Future targets

Future targets are the BMP pathway in bone and osteoclast-osteoblast coupling factors (ephrins and semaphorins). The BMPs form another branch of the TGFβ superfamily. BMP2 and BMP4 are made by osteoblasts and stimulate osteogenesis. Although BMP2 can be made by breast cancer cells, BMP2 and BMP7 are growth-inhibitory for tumor cells and their bone metastases [47], suggesting that anabolic effects on bone may not be paramount in this context. There are 14 BMP ligands and many secreted BMP antagonists and binding proteins, such as noggin, which enhances breast cancer bone metastases [56]. Contrary effects of individual BMPs and antagonists on tumor versus bone make this a complex and unpredictable area of metastasis study, despite the anabolic actions of BMPs on bone.

During normal bone homeostasis, mineral removal (by osteoclasts) and formation (by osteoblasts) are kept in balance by incompletely understood coupling mechanisms involving local signaling between the two bone cell types. Two candidate pathways involve ephrins and semaphorins. For example, osteoclasts express ephrin B2, which stimulates osteoblasts via binding to the EphB4 receptor to stimulate new bone formation [57]. The pathway is dysregulated in the bone of patients with MM [58]. Osteoclasts may also express semaphorin 4D, which suppresses bone formation via receptors on nearby osteoblasts to oppose new bone while resorption is ongoing [59]. Osteocytes may also regulate coupling in bone [6], but it is unknown whether these cells are responsive to bone-anabolic agents. The positive and negative coupling pathways offer targets for novel drugs to stimulate bone formation in osteoporotic patients. Such drugs have obvious potential in the treatment of breast cancer bone metastases. Coupling factors represent a young area of research in bone, where additional pathways may be found that provide targets for the future treatment of osteolytic bone metastases in patients with advanced breast cancer.

## Conclusions

Breast cancer bone metastasis and MM are very different tumor types but share an affinity for advanced disease to grow in bone. MM growth is accompanied by suppression of osteoblastic new formation and increased osteolytic bone destruction. In myeloma, several agents with bone-anabolic activities successfully inhibit tumor growth. Factors secreted from mature, bone-synthesizing osteoblasts may also have anti-tumor activity against metastatic cancer. Agents already in use against MM (such as bortezomib and other PIs) or for osteoporosis (strontium ranelate) may be effective, low-toxicity treatments for breast cancer bone metastases. Other agents in clinical trials - in particular, neutralizing antibodies against sclerostin and DKK1 and activin A-blocking reagents - could show similar efficacy in bone metastasis-specific clinical settings.

## Authors’ original submitted files for images

Below are the links to the authors’ original submitted files for images. Authors’ original file for figure 1

## Abbreviations

AI: aromatase inhibitor
BMD: bone mineral density
BMP: bone morphogenetic protein
BOLERO-2: Breast cancer trials of Oral Everolimus-2
DKK1: secreted Wnt inhibitor, homolog of *Drosophila* dickkopf
ER: estrogen receptor
FDA: US food and drug Administration
FGF: fibroblast growth factor
FGFR: fibroblast growth factor receptor
IL: interleukin
MET: receptor for hepatocyte growth factor
MM: multiple myeloma
mTOR: mammalian target of rapamycin
OPG: osteoprotegerin
PI: proteasome inhibitor
PTH: parathyroid hormone
RANKL: receptor activator of nuclear factor kappa B ligand
SERM: selective estrogen receptor modulator
TGFβ: transforming growth factor beta
VEGFR: vascular endothelial growth factor receptor
WNT: wingless (in *Drosophila*) signaling pathway
