# Supplementary figures and images for: Role of bone-anabolic agents in the treatment of breast cancer bone metastases
Source: Breast Cancer Res. 2014 Dec 31;16:484. doi: 10.1186/s13058-014-0484-9 (PMC4429670; doi:10.1186/s13058-014-0484-9)

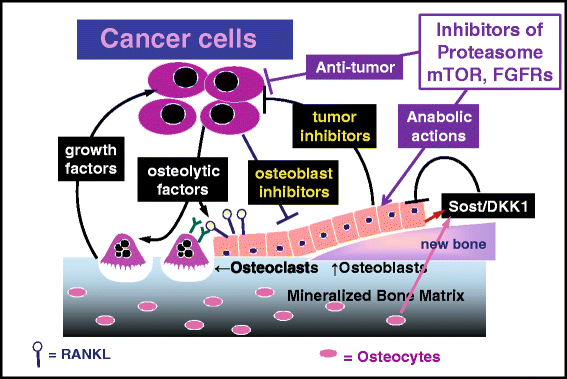

Supplement: Supplementary file 1 — Authors’ original file for figure 1 [file 13058_2014_484_MOESM1_ESM.gif]
